# Ki-67 correlations in breast cancer

**DOI:** 10.25122/jml-2025-0119

**Published:** 2025-08

**Authors:** Ruxandra Vatavu, Ana Maria Dumitrescu, Cristinel Ionel Stan, Ana Maria Haliciu, Anca Sava

**Affiliations:** 1 Doctoral School, Grigore T. Popa University of Medicine and Pharmacy, Iasi, Romania; 2 Department of Morpho-Functional Sciences I, Grigore T. Popa University of Medicine and Pharmacy, Iasi, Romania; 3 Department of Pathology, Prof. Dr. Nicolae Oblu Emergency Clinical Hospital, Iasi, Romania

**Keywords:** breast cancer, Ki-67, HER2, brain metastases, immunohistochemistry

## Abstract

Brain metastases from breast cancer represent a serious complication, associated with reduced survival and impaired quality of life. Increased patient survival and the limited ability of the blood-brain barrier to be crossed by systemic therapies have led to a rising incidence of these lesions. The molecular profile of metastases may differ from that of the primary tumor in approximately 29% of cases, significantly influencing the choice of targeted treatment. In this retrospective study, we included 100 women who underwent craniotomy for breast cancer brain metastases between 2015 and 2020 at the Prof. Dr. Nicolae Oblu Neurosurgery Clinic, Iași. We recorded demographics (age, residence), latency from primary diagnosis to brain metastasis, and MRI features (number, location, edema, hemorrhage). Histopathology and immunohistochemistry included GATA3, CK5/6, ER, PR, HER2, and Ki-67 using standardized protocols. ER/PR positivity was defined as ≥1% nuclear staining; HER2 was scored 0–3+ per ASCO/CAP; Ki-67 was reported as a percentage index. The most frequent metastatic subtypes were HER2-positive (32%) and triple-negative (25%). The mean Ki-67 index was 48.2% and showed a significant inverse correlation with the time from primary breast cancer diagnosis to brain metastasis (r = –0.57; P < 0.001). Higher Ki-67 values were associated with hemorrhagic lesions, while lower values occurred in solitary metastases. Patients receiving hormonal therapy had longer median survival (29.5 months) compared to those receiving targeted therapy (11.9 months; P < 0.001). Immunohistochemical profiling of brain metastases from breast cancer, focusing on ER, PR, HER2, and Ki-67, revealed specific correlations between tumor proliferation, time to metastasis, and neuroimaging features such as hemorrhage and lesion location. HER2-positive and triple-negative subtypes showed higher brain metastatic potential and poorer outcomes with targeted therapy, while luminal tumors responded better to hormonal treatment. The inverse correlation between Ki-67 and metastasis latency, as well as its association with aggressive imaging phenotypes, represents an original contribution of this study, underscoring the need for tailored therapeutic strategies based on combined pathological and imaging data.

## INTRODUCTION

Breast cancer is the most common malignancy in women and, despite therapeutic advances that have improved overall survival, brain metastases remain a severe complication with a major impact on prognosis and quality of life [1,2]. The incidence of breast cancer brain metastases has risen in recent years, primarily due to prolonged patient survival and the limited ability of the blood–brain barrier to be crossed by conventional systemic therapies [2,3]. Clinically, symptoms can vary, including headache, seizures, or cognitive and balance disturbances, and prognosis largely depends on the molecular subtype of the primary tumor, with triple-negative cases faring worst and HER2-positive cases showing relatively better outcomes [4]. While differentiating a primary brain neoplasm from a breast cancer metastasis is usually straightforward, conclusively establishing the adenocarcinomatous breast origin may require a complex panel of immunohistochemical markers, especially when clinical history is incomplete [5].

Immunohistochemistry (IHC) has two essential roles in this setting. First, it confirms mammary origin using markers such as GATA-binding protein 3 (GATA3), mammaglobin, and gross cystic disease fluid protein-15 (GCDFP-15), among which GATA3 shows superior sensitivity. Second, IHC molecularly characterizes brain metastases by assessing estrogen receptor (ER) and progesterone receptor (PR) expression, human epidermal growth factor receptor 2 (HER2) status, and the Ki-67 proliferation index [6,7]. This evaluation is clinically consequential: approximately 29% of patients demonstrate discordance in ER/PR/HER2 status between the primary tumor and brain metastasis, directly impacting targeted therapy selection [8,9]. Epidemiologically, about 31% of patients with HER2-positive metastatic breast cancer and 32% with the triple-negative subtype develop brain metastases—both higher than in hormone receptor–positive, HER2-negative (HR+/HER2–) disease [10].

Additionally, correlations between lesion location, number of lesions, time to brain metastasis, and immunohistochemical profile have been reported in recent studies from the past five years [2,11,12]. Specifically, HER2-positive and triple-negative tumors show a greater likelihood of multiple brain metastases and involvement of specific intracranial sites. At the same time, high Ki-67 indices are associated with shorter intervals from primary diagnosis to brain metastasis and with aggressive imaging features such as hemorrhage or edema [11,12].

Accordingly, this article aims to synthesize and analyze the immunohistochemical implications of breast cancer brain metastases, focusing on confirming breast origin with specific markers, molecular characterization of hormone receptors and HER2/neu in metastases, evaluation of discordance with primary tumors, and the impact of these findings on therapeutic and prognostic decision-making. A better understanding of these aspects will guide the optimization of multimodal treatment strategies, substantially improving patient prognosis and quality of life.

## MATERIAL AND METHODS

This retrospective study included 100 women treated at the Prof. Dr. Nicolae Oblu Neurosurgery Clinic in Iași, Romania, who underwent craniotomy for breast cancer brain metastases (for diagnostic or therapeutic purposes) between 2015 and 2020. Electronic medical records were reviewed to obtain demographic data (age at brain metastasis diagnosis), the interval from initial breast cancer diagnosis to brain metastasis, morphologic characteristics (lesion location and histopathologic subtype), and immunohistochemical profiles.

The timing of brain metastasis onset was defined as the date of the first radiologic evidence of intracranial disease on brain magnetic resonance imaging (MRI), whether performed in response to neurological symptoms or during scheduled surveillance—whichever occurred first.

For intraoperative cytopathology, smears were prepared by gently compressing 1–2 mm^3^ of tumor tissue between two clean, dry slides, followed by 1% toluidine blue staining. Cytology confirmed brain metastasis in all cases. The remaining tissue was formalin-fixed, paraffin-embedded, and sectioned at 4 μm for hematoxylin and eosin (H&E) staining. Two pathologists independently reviewed all slides and classified tumors according to the World Health Organization (WHO) Classification of Tumors of the Breast [13].

IHC employed antibodies against cytokeratin 5/6 (CK5/6) and mammaglobin (to support breast origin), ER, PR, and Ki-67 (to assess hormone receptor expression and proliferation), using EnVision™+ (Dako) and UltraVision Quanto (Thermo Scientific) systems. Antigen retrieval was performed in citrate buffer (pH 6) at 95 °C, with 3,3′-diaminobenzidine (DAB) as the chromogen. ER and PR positivity were defined as ≥1% tumor nuclei stained; Ki-67 was reported as a percentage index. HER2 was scored 0–3+ according to the American Society of Clinical Oncology/College of American Pathologists (ASCO/CAP) guidelines.

MRI examinations were performed on a 1.5-tesla (T) scanner (Siemens Magnetom Avanto). The protocol included T1-weighted spin-echo, T2-weighted fast spin-echo, fluid-attenuated inversion recovery (FLAIR), susceptibility-weighted imaging (SWI), diffusion-weighted imaging (DWI), and contrast-enhanced T1-weighted sequences acquired in axial, coronal, and sagittal planes. From the digital archive, we extracted lesion number, location, extent of edema, and presence of hemorrhage. Statistical analyses were conducted in IBM SPSS Statistics, version 27. Categorical variables were compared using chi-square (χ^2^) tests; continuous variables were compared with Student’s *t* test or the Mann–Whitney U test, as appropriate. Survival was analyzed using Kaplan–Meier methods with the log-rank test. The significance threshold was set at α = 0.05.

## RESULTS

### Demographic characteristics and clinical presentation

The study cohort comprised 100 women with breast cancer brain metastases. The mean age at diagnosis of brain metastases was 54.2 years (range: 32–78 years). Fifty-one percent were younger than 55 years, and 49% were 55 years or older; 55% resided in rural areas versus 45% in urban settings (Table 1). For comparative purposes, patients were grouped as younger than 55 years or 55 years and older, reflecting the approximate transition from predominantly premenopausal to predominantly postmenopausal status, which can influence breast cancer biology, hormonal environment, and therapeutic response [14-16].

**Table 1:** Demographic characteristics of the study cohort

Variable	*n* (%) or mean ± SD	Range / Notes
Number of patients	100	—
Age at BM diagnosis (years)	54.2 ± 10.3	32–78
Age group <55 years	51 (51%)	—
Age group ≥55 years	49 (49%)	—
Rural residence	55 (55%)	—
Urban residence	45 (45%)	—
Number of brain metastases	1.97 ± 1.1	1–5
Single metastasis	47 (47%)	—
≥3 metastases	28 (28%)	—
Most common symptoms	Confusion (22%), trigeminal neuralgia (11%), hemianesthesia (11%), hemianopsia (10%)	—

**Figure 1:**
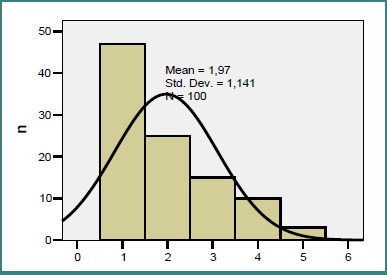
Histogram of the number of metastases

The number of metastases per patient ranged from 1 to 5 (mean 1.97; median 2; skewness 0.975), with 47% of patients presenting a single lesion and 28% having three or more metastases (Figure 1). At clinical presentation, the most frequent neurological symptoms were confusion (22%), trigeminal neuralgia (11%), hemianesthesia (11%), and hemianopsia (10%).

### Radiologic features

Contrast-enhanced brain MRI revealed 47% of cases with solitary lesions, multiple lesions in 24%, necrotic features in 9%, and leptomeningeal invasion in 7%. Perilesional edema was observed in 62% of patients, and hemorrhagic features were identified in 18% of cases. The mean interval from primary breast cancer diagnosis to detection of brain metastases was 17.03 months (median 14 months; skewness 0.949).

### Lesion location and molecular subtypes

Metastases were most commonly located in the parietal lobe (61%), followed by the Gasserian ganglion (11%), frontal lobe (6%), temporal lobe (5%), occipital lobe (4%), cerebellum (4%), optic nerve (2%), basal ganglia (2%), thalamus (2%), and corpus callosum (3%). Although metastases to the Gasserian (trigeminal) ganglion are rarely reported in the literature, the relatively high proportion observed in our study may be explained by the fact that the Prof. Dr. Nicolae Oblu Emergency Clinical Hospital is a national referral center for neurosurgery, with extensive experience in treating skull base lesions. In addition, the close anatomical relationship between the middle cranial fossa dura and the trigeminal ganglion may facilitate metastatic spread via perineural or leptomeningeal extension in advanced disease.

**Figure 2:**
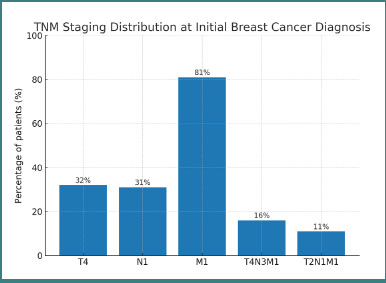
TNM staging distribution at initial breast cancer diagnosis among patients with brain metastases

The primary breast tumors most often arose in the upper outer quadrant (27%), followed by central (20%), retroareolar (16%), lower outer (13%), upper inner (12%), and lower inner (12%) locations. At initial staging, tumor–node–metastasis (TNM) categories were predominantly T4 (32%) and N1 (31%), with distant metastasis present (M1) in 81% of patients; 16% had T4N3M1 disease and 11% had T2N1M1 disease (Figure 2). Histopathologic subtypes of brain metastases included HER2-positive (32%), triple-negative (25%), Luminal B (19%), Luminal A (17%), metaplastic (4%), and mucinous (3%) (Table 2).

**Table 2:** Pathological and molecular characteristics of brain metastases from breast cancer according to staging

Variable	*n* (%)	Correlations with other variables
Histopathologic subtype		
– HER2-positive	32 (32%)	More frequent in patients <55 years; higher Ki-67 values
– Triple-negative	25 (25%)	Shorter time to brain metastasis; frequent hemorrhagic MRI features
– Luminal B	19 (19%)	Intermediate Ki-67 values
– Luminal A	17 (17%)	Longer time to brain metastasis; lower Ki-67 values
– Metaplastic	4 (4%)	Aggressive MRI appearance
– Mucinous	3 (3%)	Rare; lower proliferation index
Mean Ki-67 (%)	48.2 ± 27.5	Inversely correlated with time to metastasis (P < 0.001)
ER-positive	53 (53%)	Associated with Luminal subtypes
PR-positive	32 (32%)	Often co-expressed with ER
HER2-positive	46 (46%)	Includes HER2+ and Luminal B subtypes
Triple-negative (ER-/PR-/HER2-)	32 (32%)	Higher proliferation; frequent hemorrhage

The bar chart shows the percentage of patients in each staging category: T4 (32%), N1 (31%), M1 (81%), T4N3M1 (16%), and T2N1M1 (11%). Percentages are displayed above each bar for clarity.

### Cellular proliferation and correlations

The Ki-67 proliferation index ranged from 5% to 98%, with a mean of 48.20% and a median of 46.50%. The skewness of the distribution was 0.249, indicating approximate normality and supporting the use of parametric significance tests for continuous variables (Table 3).

**Table 3:** Descriptive statistical indicators for Ki-67 (%)

Indicator	Value
*n*	100
Mean	48.20
Median	46.50
Standard deviation	27.55
Variance	57.16
Skewness	0.249
Standard error of skewness	0.241
Minimum	5
Maximum	98
25^th^ percentile	25.00
50^th^ percentile	46.50
75^th^ percentile	73.75

Ki-67% showed a weak inverse correlation with patient age (r = –0,154; *P* = 0,126), indicating that only 15.4% of the variability in proliferation index could be attributed to age-related decreases, though this trend did not reach statistical significance.

**Figure 3:**
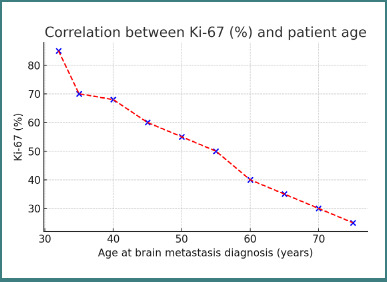
Correlation between Ki-67 (%) and patient age

**Figure 4:**
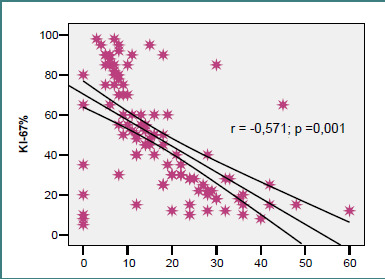
Correlation of KI-67% with duration of metastasis

Figure 3 is a scatter plot showing the relationship between Ki-67 proliferation index and patient age at the time of brain metastasis diagnosis. A weak inverse trend is observed (r = –0.154; *P* = 0.126), suggesting slightly lower proliferation in older patients, though not statistically significant. There was a statistically significant moderate inverse correlation between Ki-67% and the time from primary diagnosis to brain metastasis (r = –0.571; *P* = 0.001; Figure 4), indicating that patients whose metastases appeared later tended to have lower proliferation indices—57.1% of cases with longer latency exhibited reduced Ki-67 levels.

### Correlation of Ki-67% with metastasis location

**Figure 5:**
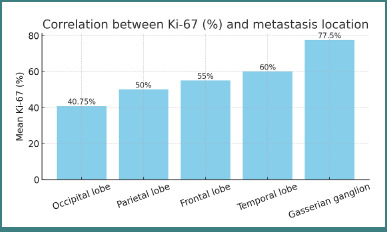
Correlation between Ki-67 (%) and metastasis location

Analysis of Ki-67% by anatomical site revealed significant differences (Figure 5):

- The lowest mean Ki-67 index (40.75%) was observed in metastases located in the occipital lobe (*P* = 0.038).
- The highest mean Ki-67 index (77.50%) occurred in metastases involving the Gasserian (trigeminal) ganglion (*P* = 0.011).

These findings suggest that tumor proliferation rates vary by intracranial location, potentially reflecting microenvironmental influences on tumor biology.

Bar chart illustrating mean Ki-67 values according to anatomical site of brain metastases. The lowest mean value (40.75%) occurred in occipital lobe metastases, while the highest (77.50%) was seen in Gasserian ganglion metastases.

### Correlation of Ki-67% with radiologic features

**Figure 6:**
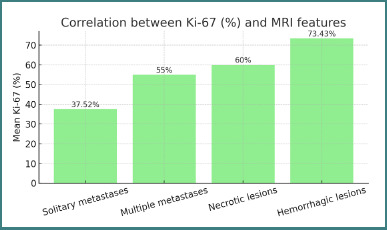
Correlation between Ki-67 (%) and MRI features

When stratified by MRI presentation (Figure 6), Ki-67 proliferation indices differed significantly across radiologic patterns:

- Patients with solitary brain metastases exhibited the lowest mean Ki-67% (37.52%; *P* = 0.001).
- Patients whose lesions showed hemorrhagic features on MRI had the highest mean Ki-67% (73.43%; *P* = 0.001).

These results indicate that higher proliferative activity is associated with hemorrhagic metastases, whereas solitary lesions tend to display lower Ki-67 indices.

Figure 6 compares mean Ki-67 values across different radiologic presentations. Solitary metastases showed the lowest mean Ki-67 (37.52%), whereas hemorrhagic lesions exhibited the highest (73.43%). In patients with brain metastases originating from breast cancer, the predominant treatment was targeted (36%), followed by hormonal therapy (23%) and chemotherapy (20%)

### Treatment and combinations

Chemotherapy and radiotherapy were administered uniformly by age group, while targeted therapy predominated among patients under 55 years of age (66.7%), and hormonal therapy (56.5%) and radiotherapy (54.5%) were more common in those aged 55 years and older (*P* = 0.025).

Patients who received targeted therapy had the shortest mean duration of metastasis (11.89 months), and those with hormonal therapy had the longest (29.48 months; *P* = 0.001). Regarding Ki-67, the lowest mean value (31.33%) was recorded in patients without treatment, and the highest (87.0%) in those with immunotherapy (*P* = 0.001).

### BRCA status

Of 100 patients, 20% were positive for BRCA1 and 11% for BRCA2 (*BRCA*: breast cancer susceptibility gene). In the BRCA1-positive group, 50% were over 55 years of age (*P* = 0.077), 70% were from rural areas (*P* = 0.302), 50% had ≥3 metastases (*P* = 0.003), 35% had parietal metastases (*P* = 0.029) and 40% had evidence of hemorrhage on imaging (*P* = 0.003).

## DISCUSSION

Our results clearly highlight the clinical and molecular heterogeneity of breast cancer brain metastases, emphasizing the determinant role of the prognostic factors analyzed. The balanced age distribution in our cohort (51% vs. 49%) and predominance of patients from rural areas (55%) suggest that differences in access to specialized oncologic care, rather than age itself, significantly influence the incidence of brain metastases, as observed in population-based analyses showing delayed diagnosis and suboptimal treatment initiation in rural settings [17-19]. Imaging analysis revealed a high proportion of solitary metastases (47%) and a predominance of parietal lobe involvement (61%), which may be explained by the parietal lobe’s large cortical surface, rich vascular supply via the middle cerebral artery, and its status as a watershed zone predisposing to metastatic seeding; similar spatial distribution patterns have been documented in recent MRI mapping studies of breast cancer brain metastases, reporting parietal involvement rates between 55–63% [20,21]. This anatomical predilection is thought to be influenced by hemodynamic characteristics and venous drainage patterns that facilitate tumor cell deposition, particularly in aggressive molecular subtypes such as HER2-positive and triple-negative disease [22,23]. The molecular profile of brain metastases in our cohort revealed a predominance of HER2-positive (32%) and triple-negative (25%) subtypes, consistent with evidence that these phenotypes exhibit higher neurotropism due to limited central nervous system penetration of systemic therapies and intrinsic biological aggressiveness [23-25]. Our finding of a mean Ki-67 index of 48.2% and its significant inverse correlation with the interval from primary diagnosis to brain metastasis supports previous reports that high proliferative activity accelerates CNS dissemination in breast cancer [26]. Ki-67 also showed notable associations with lesion location and imaging characteristics: higher values were observed in Gasserian ganglion metastases and hemorrhagic lesions, while lower values characterized occipital lobe lesions and solitary metastases, paralleling recent neuropathological studies linking tumor microenvironment and vascular fragility to proliferation rates and hemorrhagic propensity [27]. The relationship between Ki-67 and radiologic phenotype is particularly relevant, as high proliferative indices have been proposed as a predictive marker for hemorrhagic presentation in brain metastases across multiple primaries, suggesting a potential role for Ki-67 as both a histopathological and imaging biomarker [27]. The survival advantage seen with hormonal therapy in luminal subtypes in our study aligns with pharmacokinetic evidence showing better intracranial bioavailability of endocrine agents compared to monoclonal antibodies, as well as with clinical outcome data from recent real-world registries [28,29]. Furthermore, BRCA1/2 mutation carriers in our cohort exhibited a higher number of brain lesions and more frequent hemorrhagic features, consistent with recent genomic studies describing an association between homologous recombination deficiency, genomic instability, and vascular fragility in metastatic breast cancer [30]. Collectively, these findings underscore the importance of integrating clinical, pathological, and imaging parameters in the management of breast cancer brain metastases, and they provide contemporary evidence that Ki-67 is a multidimensional prognostic marker whose value extends beyond simple proliferation assessment to encompass locoregional biology and radiologic behavior.

## CONCLUSION

The analysis of correlations between the Ki-67 proliferation index and the pathological and radiological characteristics of breast cancer brain metastases showed that higher Ki-67 values were associated with aggressive imaging features, particularly the presence of hemorrhage, which may reflect tumor vessel fragility and rapid growth dynamics. Metastases located in the Gasserian ganglion exhibited higher Ki-67 values, while lower values were observed in occipital lobe metastases and solitary lesions, suggesting a biological behavior that varies according to cerebral location.

Immunohistochemical profiling, including ER, PR, HER2, and Ki-67, correlated with clinical and imaging data, allowing a more precise characterization of breast cancer brain metastases. In the studied cohort, HER2-positive and triple-negative subtypes were more frequent and displayed higher Ki-67 values, associated with shorter intervals from primary tumor diagnosis to brain metastasis onset. Luminal tumors showed lower Ki-67 values and longer brain metastasis–free intervals. These findings may support the adaptation of therapeutic strategies and monitoring programs according to molecular profile and tumor proliferation rate.

The correlation of Ki-67 values with imaging phenotypes suggests its utility not only in histopathological evaluation but also in anticipating the radiological behavior of brain metastases, including the risk of hemorrhage, multiplicity, and specific localization. Integrating this information may contribute to the personalization of surgical planning, radiotherapy strategy, and systemic therapy selection, as well as to the exploration of advanced imaging approaches for non-invasive estimation of tumor characteristics.

## References

[1] Sung H, Ferlay J, Siegel RL, Laversanne M, Soerjomataram I, Jemal A (2021). Global Cancer Statistics 2020: GLOBOCAN estimates of incidence and mortality worldwide for 36 cancers in 185 countries. CA Cancer J Clin.

[2] Raghavendra AS, Ibrahim NK (2024). Breast Cancer Brain Metastasis: A Comprehensive Review. JCO Oncol Pract.

[3] Zimmer AS, Van Swearingen AED, Anders CK (2020). HER2-positive breast cancer brain metastasis: A new and exciting review. Cancer Rep (Hoboken).

[4] Sperduto PW, Mesko S, Li J, Cagney D, Aizer A, Lin NU (2020). Estrogen/progesterone receptor and HER2 discordance between primary tumor and brain metastases in breast cancer and its effect on treatment and survival. Neuro Oncol.

[5] Ding Q, Huo L, Peng Y, Yoon EC, Li Z, Sahin AA (2022). Immunohistochemical Markers for Distinguishing Metastatic Breast Carcinoma from Other Common Malignancies: Update and Revisit. Semin Diagn Pathol.

[6] Cimino-Mathews A (2021). Novel uses of immunohistochemistry in breast pathology: interpretation and pitfalls. Mod Pathol.

[7] Hulsbergen AFC, Claes A, Bashir S, Kavouridis VK, Engel DC, Broekman MLD (2020). Subtype switching in breast cancer brain metastases: a multicenter analysis. Neuro Oncol.

[8] Kotecha R, Tonse R, Rubens M, McDermott MW, Odia Y, Appel H (2021). Systematic review and meta-analysis of breast cancer brain metastasis and primary tumor receptor expression discordance. Neurooncol Adv.

[9] Kuksis M, Gao Y, Tran W, Hoey C, Kiss A, Komorowski AS (2021). The incidence of brain metastases among patients with metastatic breast cancer: a systematic review and meta-analysis. Neuro Oncol.

[10] Hackshaw MD, Danysh HE, Henderson M, Wang E, Tu N, Islam Z (2021). Prognostic factors of brain metastasis and survival among HER2-positive metastatic breast cancer patients: a systematic literature review. BMC Cancer.

[11] Michel A, Dinger T, Darkwah Oppong M, Rauschenbach L, Deuschl C, Ahmadipour Y (2022). Radiographic markers of breast cancer brain metastases: relation to clinical characteristics and postoperative outcome. Acta Neurochir (Wien).

[12] Mohammadi M, Mohammadi S, Hadizadeh H, Olfati M, Moradi F, Tanzifi G, Ghaderi S (2024). Brain metastases from breast cancer using magnetic resonance imaging: A systematic review. J Med Radiat Sci.

[13] Tan PH, Ellis I, Allison K, Brogi E, Fox SB, Lakhani S (2020). WHO Classification of Tumours Editorial Board. The 2019 World Health Organization classification of tumours of the breast. Histopathology.

[14] Lega IC, Fine A, Antoniades ML, Jacobson M (2023). A pragmatic approach to the management of menopause. CMAJ.

[15] Early Breast Cancer Trialists' Collaborative Group (EBCTCG) (2022). Aromatase inhibitors versus tamoxifen in premenopausal women with oestrogen receptor-positive early-stage breast cancer treated with ovarian suppression: a patient-level meta-analysis of 7030 women from four randomised trials. Lancet Oncol.

[16] Nishimura R, Osako T, Okumura Y, Nakano M, Otsuka H, Fujisue M, Arima N (2022). Triple Negative Breast Cancer: An Analysis of the Subtypes and the Effects of Menopausal Status on Invasive Breast Cancer. J Clin Med.

[17] Bhatia S, Landier W, Paskett ED, Peters KB, Merrill JK, Phillips J (2022). Rural-Urban Disparities in Cancer Outcomes: Opportunities for Future Research. J Natl Cancer Inst.

[18] LeBlanc G, Lee I, Carretta H, Luo Y, Sinha D, Rust G (2022). Rural-Urban Differences in Breast Cancer Stage at Diagnosis. Womens Health Rep (New Rochelle).

[19] Adams SA, Babatunde OA, Zahnd WE, Hung P, Wickersham KE, Bell N, Eberth JM (2025). An Investigation of Travel Distance and Timeliness of Breast Cancer Treatment Among a Diverse Cohort in the United States. Int J Environ Res Public Health.

[20] Lin B, Huang D, Yang X, Zhang Y, Gang F, Du XB (2020). Distribution of brain metastases: low-risk metastasis areas may be avoided when treating with whole-brain radiotherapy. Cancer Imaging.

[21] Cardinal T, Pangal D, Strickland BA, Newton P, Mahmoodifar S, Mason J (2021). Anatomical and topographical variations in the distribution of brain metastases based on primary cancer origin and molecular subtypes: a systematic review. Neurooncol Adv.

[22] Bao H, Ren P, Liang X, Lai J, Bai Y, Liu Y (2024). The Spatial Distribution of Brain Metastasis Is Determined by the Heterogeneity of the Brain Microenvironment. Hum Brain Mapp.

[23] Sun H, Xu J, Dai S, Ma Y, Sun T (2023). Breast cancer brain metastasis: Current evidence and future directions. Cancer Med.

[24] Zimmer AS (2021). "Triple-Negative Breast Cancer Central Nervous System Metastases From the Laboratory to the Clinic". Cancer J.

[25] Jiaxin C, Jinmei Z, Huiqiang Z, Xuexue W, Xiaobo W, Shaohua Z (2022). Conversion of ER, PR, HER2 and Ki-67 and prognosis in breast cancer metastases to the brain. Front Neurol.

[26] Farina J, Angelico G, Vecchio GM, Salvatorelli L, Magro G, Puzzo L (2023). Brain Metastases from Breast Cancer Histologically Exhibit Solid Growth Pattern with at Least Focal Comedonecrosis: A Histopathologic Study on a Monocentric Series of 30 Cases. Diagnostics (Basel).

[27] Curtaz CJ, Kiesel L, Meybohm P, Wöckel A, Burek M (2022). Anti-Hormonal Therapy in Breast Cancer and Its Effect on the Blood-Brain Barrier. Cancers (Basel).

[28] Chen Q, Xiong J, Ma Y, Wei J, Liu C, Zhao Y (2022). Systemic treatments for breast cancer brain metastasis. Front Oncol.

[29] Galland L, Roussot N, Desmoulins I, Mayeur D, Kaderbhai C, Ilie S (2023). Clinical Utility of Genomic Tests Evaluating Homologous Recombination Repair Deficiency (HRD) for Treatment Decisions in Early and Metastatic Breast Cancer. Cancers (Basel).

[30] Linville RM, Maressa J, Guo Z, Chung TD, Farrell A, Jha R (2023). A tissue-engineered model of the blood-tumor barrier during metastatic breast cancer. Fluids Barriers CNS.

